# The Biochemical and Genetic Architecture of Geographic Atrophy: The Role of the FHL-1/CFH Axis and the Paradigm of RNA Interference Therapeutics

**DOI:** 10.3390/biomedicines14081809

**Published:** 2026-08-12

**Authors:** Victor Chong

**Affiliations:** 1 UCL Institute of Ophthalmology, London EC1V 9EL, UK; victor@eretina.org; 2 Myocross Vision LLC, 11800 Veterans Pkwy, Reno, NV 89521, USA

**Keywords:** age-related macular degeneration, geographic atrophy, complement biology, CFH, FHL-1, siRNA

## Abstract

Geographic atrophy (GA) represents the advanced, non-neovascular (dry) form of age-related macular degeneration (AMD), a chronic, progressive, and currently irreversible neurodegenerative disease of the retina. The clinical consequences of GA are severe; it is characterized by the insidious, expanding loss of the retinal pigment epithelium (RPE), the overlying photoreceptors, and the underlying choriocapillaris. This state of complete RPE and outer retinal atrophy (cRORA) permanently destroys the neural architecture required for high-acuity central vision. For decades, the pathophysiological etiology of geographic atrophy was framed principally in terms of cumulative oxidative stress, lipid peroxidation, and cellular senescence. However, the foundational understanding of AMD pathophysiology changed substantially following the landmark genomic discoveries published in 2005. Multiple independent genome-wide association studies (GWAS) linked specific single-nucleotide polymorphisms in the *CFH* gene to a substantially increased risk of developing AMD. The *CFH* gene encodes Complement Factor H (FH) and its alternative splice variant, Factor H-like protein 1 (FHL-1), which are the primary soluble regulators of the alternative complement pathway. This genetic discovery established GA not merely as a disease of metabolic wear-and-tear, but fundamentally as an immunologic disorder driven by the chronic dysregulation of the innate immune system. With the rapid emergence and clinical validation of targeted gene-silencing technologies, particularly small interfering RNA (siRNA) and antisense oligonucleotides, there is substantial scientific and pharmaceutical interest in modulating the complement cascade at the post-transcriptional level. This narrative review examines the structural biology, spatial partitioning, and pathophysiological roles of the FHL-1/CFH axis in GA focusing on the possibilities of using siRNA as a new potential therapy for GA.

## 1. Introduction

Geographic atrophy (GA) represents the advanced, non-neovascular (dry) form of age-related macular degeneration (AMD), a chronic, progressive, and currently irreversible neurodegenerative disease of the retina. As populations age across the developed world, the incidence of GA has surged, currently affecting an estimated 8 million individuals globally and standing as a leading cause of untreatable central blindness, particularly among Caucasian populations. The clinical consequences of geographic atrophy are severe; it is characterized by the insidious, expanding loss of the retinal pigment epithelium (RPE), the overlying photoreceptors, and the underlying choriocapillaris [1]. These atrophic lesions typically originate in the parafoveal region and expand to encompass the fovea, ultimately culminating in severe visual impairment and a profound loss of patient independence [1]. This state of complete RPE and outer retinal atrophy (cRORA) permanently destroys the neural architecture required for high-acuity central vision [2].

For decades, the pathophysiological etiology of geographic atrophy was framed principally in terms of cumulative oxidative stress, lipid peroxidation, and cellular senescence. The retina is one of the most metabolically active tissues in the human body, and the RPE is tasked with the continuous burden of phagocytosing shed photoreceptor outer segments while neutralizing reactive oxygen species generated by constant light exposure. It was long hypothesized that GA was simply the end-stage failure of these metabolic support systems, exacerbated by age. However, the foundational understanding of AMD pathophysiology changed substantially following the landmark genomic discoveries published in 2005. Multiple independent genome-wide association studies (GWAS) linked specific single-nucleotide polymorphisms in the *CFH* gene to a substantially increased risk of developing AMD [3,4]. The *CFH* gene encodes Complement Factor H (FH) and its alternative splice variant, Factor H-like protein 1 (FHL-1), which are the primary soluble regulators of the alternative complement pathway [5].

This genetic discovery established geographic atrophy not merely as a disease of metabolic wear-and-tear, but fundamentally as an immunologic disorder driven by the chronic dysregulation of the innate immune system [6]. The complement system, traditionally viewed as a frontline defense mechanism against invasive microbial pathogens, was suddenly implicated as a driver of host tissue damage in the aging eye [7]. Subsequent genetic analyses have robustly confirmed this paradigm, identifying an entire network of risk variants clustered within genes encoding various complement components, including complement component 3 (C3), complement component 5 (C5), complement factor B (CFB), complement factor I (CFI), and a suite of Factor H-related proteins [6].

When these delicate regulatory mechanisms fail—often due to genetic variants in the *CFH* locus that impair the function of FH and FHL-1—the alternative pathway amplification loop proceeds unchecked on the surfaces of host tissues, particularly along the critical RPE–Bruch’s membrane interface [8]. This unchecked biochemical cascade leads to chronic low-grade inflammation, the accumulation of complement breakdown products within extracellular deposits known as drusen, and the eventual necrotic and apoptotic death of the RPE cells [5].

This proposition requires careful framing. AMD is polygenic and multifactorial, and complement dysregulation is one axis within it rather than a complete account of the disease. Large genome-wide analyses identify independent association signals at more than thirty loci, of which the *CFH* region at 1q31.3 and the *ARMS2/HTRA1* region at 10q26 carry the largest effects; the 10q26 locus contains no complement gene at all [9]. Aging itself, cigarette smoking, dietary and lipid-handling factors, mitochondrial and lysosomal dysfunction within the RPE, oxidative stress, extracellular matrix remodeling, and choriocapillaris dropout all contribute, and their relative weight differs between individuals. The argument advanced in this review is correspondingly bounded: that the FHL-1/CFH axis is a dominant and pharmacologically tractable mechanistic axis in a substantial subset of patients, not that it is a sufficient explanation for geographic atrophy in all of them.

With the rapid emergence and clinical validation of targeted gene-silencing technologies, particularly small interfering RNA (siRNA) and antisense oligonucleotides, there is substantial scientific and pharmaceutical interest in modulating the complement cascade at the post-transcriptional level. This review examines the structural biology, spatial partitioning, and pathophysiological roles of the FHL-1/CFH axis in geographic atrophy. Furthermore, it explores the mechanistic basis of regulatory failure, critically evaluates the theoretical and active clinical applications of siRNA therapeutics targeting this specific pathway, and delineates why direct silencing of FHL-1 is contraindicated while indirect, network-level RNA interference represents a more defensible therapeutic direction.

## 2. Scope and Methods

This article is a narrative, focused review and is not a systematic review. Its scope was defined in advance and deliberately restricted to two questions: the role of the FHL-1/CFH regulatory axis at the retinal pigment epithelium–Bruch’s membrane interface, and whether RNA interference can be applied rationally to that axis. Comprehensive reviews of AMD pathogenesis, of complement biology in general, and of the full therapeutic pipeline in geographic atrophy are already available and are not duplicated here.

Literature was identified by searching PubMed/MEDLINE and Web of Science from January 1990 to May 2026 using combinations of the terms *complement factor H, CFH, factor H-like protein 1, FHL-1, factor H-related protein, CFHR, Y402H, Bruch’s membrane, geographic atrophy, age-related macular degeneration, small interfering RNA, siRNA, RNA interference* and *antisense oligonucleotide*. ClinicalTrials.gov and the EU Clinical Trials Register were searched for interventional studies of complement-directed agents in geographic atrophy, and the reference lists of retrieved articles were hand-searched. English-language primary reports were eligible, together with reviews where these provided consolidated structural or epidemiological data. Preference was given to human genetic, human donor tissue, and clinical data; animal data are cited where they constitute the only available evidence, and the species limitation is stated at each point.

Article selection was made by the author on the basis of relevance to the two questions above and was not governed by a formal quality-appraisal instrument. The synthesis therefore reflects the author’s interpretation and carries the selection biases inherent to narrative review. To mitigate this, claims are graded throughout by the type of evidence supporting them—genetic association, circulating protein association, tissue-level observation, or interventional data. This grading is applied explicitly to the factor H-related proteins and is summarized for all eight proteins considered in Table 1.

## 3. The Biochemical Machinery of the Complement System

To fully appreciate the protective role of FHL-1 and the rationale for siRNA intervention, one must first intricately delineate the biochemical architecture of the complement system. The complement cascade is a phylogenetically ancient, highly conserved arm of innate immunity consisting of over 30 soluble plasma proteins, membrane-bound receptors, and regulatory factors [5]. It operates as a sophisticated proteolytic cascade designed to rapidly opsonize pathogens, recruit inflammatory cells via chemotaxis, and directly lyse foreign entities or compromised host cells via the assembly of the membrane attack complex (MAC) [5]. The cascade is initiated via three distinct but converging enzymatic routes: the classical pathway, the lectin pathway, and the alternative pathway [10].

The classical pathway is typically triggered by the binding of the C1q protein complex to antibody–antigen complexes (primarily IgM or IgG clusters) on a target surface [5]. The lectin pathway is initiated when mannose-binding lectin or ficolins recognize specific carbohydrate motifs, such as microbial sugars, on the surfaces of pathogens [10]. Both of these pathways rely on specific recognition events to trigger their respective proteolytic cascades. However, the alternative pathway (AP) does not depend on such recognition events and is the pathway most strongly implicated in geographic atrophy [6,10].

Unlike the classical and lectin pathways, the alternative pathway does not require specific pathogen recognition, antibodies, or exogenous triggers to initiate. Instead, it relies on a continuous, spontaneous state of low-level activation [11]. The central protein of the complement system, C3, contains a highly reactive internal thioester bond. In the fluid phase (plasma or interstitial fluid), this bond undergoes spontaneous hydrolysis at a slow but steady rate, converting intact C3 into the biologically active hydrolyzed form, C3(H2O) [6]. This hydrolyzed C3 molecule undergoes a conformational change that allows it to bind to Complement Factor B (CFB) [6].

Once bound to hydrolyzed C3, Factor B is exposed to cleavage by a constitutively active circulating serine protease known as Factor D (CFD) [6,10]. Factor D cleaves Factor B into a small Ba fragment and a larger Bb catalytic fragment, which remains bound to the complex, forming the initial fluid-phase C3 convertase [6]. This soluble convertase possesses the enzymatic capability to cleave additional, intact circulating C3 molecules into two distinct fragments: C3a and C3b. C3a is a small, highly potent anaphylatoxin that diffuses into the surrounding tissue, inducing localized vasodilation, increasing vascular permeability, and acting as a powerful chemoattractant for circulating immune cells such as macrophages and microglia [6,10].

The larger fragment, C3b, is the central effector molecule of the entire complement cascade. Upon cleavage, the internal thioester bond of C3b is momentarily exposed. If C3b is adjacent to a biological surface—whether a bacterial cell wall, a viral envelope, or a host cell membrane—the thioester bond rapidly forms a covalent ester or amide linkage with nucleophilic hydroxyl or amino groups on that surface [6]. If C3b fails to encounter a surface within microseconds, the thioester bond is neutralized by water, rendering the molecule inert. However, once covalently tethered to a surface, C3b serves as a platform for amplification of the complement response [6].

The C5 convertase acts on complement component 5 (C5), cleaving it into C5a and C5b [6]. C5a is an even more potent anaphylatoxin and chemoattractant than C3a, driving severe local inflammation [6,10]. The larger C5b fragment remains associated with the surface and initiates the terminal lytic pathway [6,10]. C5b recruits complement components C6, C7, and C8, which anchor the complex into the lipid bilayer of the target cell membrane [6,10]. Finally, multiple molecules of C9 (typically 10 to 16) polymerize to form a transmembrane pore known as the Membrane Attack Complex (MAC, or C5b-9) [6,10]. The insertion of the MAC destroys the selective permeability of the lipid bilayer, leading to rapid osmotic dysregulation, calcium influx, and necrotic cell lysis [6,10].

Because the alternative pathway is continuously generating highly reactive C3b that can bind indiscriminately to any nearby surface, host tissues are continuously exposed to the risk of incidental complement deposition [6,10]. To prevent autologous tissue destruction, human cells have evolved a sophisticated array of membrane-bound regulators, including Membrane Cofactor Protein (MCP, or CD46), Decay Accelerating Factor (DAF, or CD55), and CD59 (which inhibits MAC assembly) [12]. However, the specific anatomical and microenvironmental characteristics of the retina render it uniquely vulnerable to alternative pathway dysregulation, elevating the importance of soluble regulators like FHL-1 to an absolute necessity.

## 4. The Retinal Microenvironment and the Vulnerability of Bruch’s Membrane

The posterior segment of the human eye is a highly specialized anatomical structure designed to capture photons and convert them into electrochemical signals while managing a high metabolic load. The neuroretina, containing the light-sensing photoreceptors, is supported by a monolayer of highly polarized, pigmented cells known as the retinal pigment epithelium (RPE) [1]. The RPE performs myriad critical functions: it recycles visual chromophores, transports nutrients from the blood to the photoreceptors, phagocytoses continuously shed photoreceptor outer segments, and maintains the blood–retinal barrier [1].

Beneath the RPE lies the choroid, a densely vascularized tissue that supplies the oxygen and nutritional demands of the outer retina [1]. Separating the RPE from the fenestrated capillaries of the choroid (the choriocapillaris) is Bruch’s membrane [4]. Bruch’s membrane is a complex, pentalaminar extracellular matrix (ECM) composed of a central elastic layer flanked by inner and outer collagenous zones, and the basal laminas of the RPE and the choriocapillaris [1]. This membrane acts as a critical physical support structure and a semi-permeable filtration barrier, regulating the exchange of biomolecules, nutrients, and waste products between the systemic circulation and the retina.

The unique vulnerability of the retinal microenvironment to complement-mediated damage stems directly from the nature of Bruch’s membrane. Because Bruch’s membrane is an acellular extracellular matrix, it inherently lacks all intrinsic, membrane-bound complement regulators [5,12]. It does not possess CD46, CD55, or CD59 to defend itself against the spontaneous deposition of C3b [12]. Furthermore, as an extracellular matrix, it is rich in nucleophilic groups that readily accept the covalent binding of C3b generated by the alternative pathway tick-over mechanism [5].

Consequently, the protection of Bruch’s membrane from complement amplification depends on the recruitment of fluid-phase (soluble) complement regulators [5]. These soluble regulators must either be synthesized locally within the eye or delivered via the choroidal circulation and successfully permeate the layers of Bruch’s membrane [1]. The RPE is not merely a structural barrier; it is an active immunological bio-factory. In healthy states, RPE cells basally secrete an array of complement components directly into their microenvironment, including C3, Factor B, Factor I, Complement Factor H (FH), and Factor H-like protein 1 (FHL-1) [13]. This localized production contributes to crucial immune surveillance and retinal homeostasis, clearing cellular debris without inciting damaging inflammation [4,5,14].

However, the localized production of complement proteins also means that the RPE supplies the very fuel (C3 and Factor B) required for the alternative pathway amplification loop [4]. If the balance between these locally synthesized activators and the soluble regulators (FH and FHL-1) is disrupted, Bruch’s membrane becomes a primary focal point for unregulated complement deposition. Over decades, this unregulated deposition leads to the accumulation of complement breakdown products, oxidized lipids, and cellular debris between the RPE and Bruch’s membrane, forming the hallmark clinical lesions known as drusen [1]. Drusen are not inert deposits; they are inflammatory microenvironments saturated with C3, C5, and MAC, acting as a constant stimulus for local parainflammation [5,14]. The biochemical integrity of Bruch’s membrane and the survival of the RPE are therefore closely dependent on the structural and functional efficacy of the FH/FHL-1 regulatory axis, alongside the other aging-related processes that operate at this interface.

## 5. Structural Biology and Spatial Partitioning: Complement Factor H Versus FHL-1

The *CFH* gene, located on chromosome 1q32 within a region known as the Regulators of Complement Activation (RCA) gene cluster, encodes the principal soluble regulator of the alternative complement pathway [13]. Through the process of alternative mRNA splicing, this single genetic locus encodes two distinct, highly specialized complement regulatory proteins: the full-length Complement Factor H (FH) and its significantly truncated variant, Factor H-like protein 1 (FHL-1) [13].

## 6. The Molecular Architecture of FH and FHL-1

Full-length Complement Factor H is a large, heavily glycosylated plasma protein with a molecular weight of approximately 139 to 150 kDa [6,13]. It is synthesized primarily by the liver, circulating in human blood at high concentrations (approximately 300 to 500 µg/mL), but it is also produced locally by various cell types, including the RPE [4]. The FH protein is composed of a continuous string of 20 distinct globular domains known as short consensus repeats (SCRs), also referred to as complement control protein (CCP) domains [6]. Each SCR is approximately 60 amino acids in length and is stabilized by conserved disulfide bonds.

**Figure 1:**
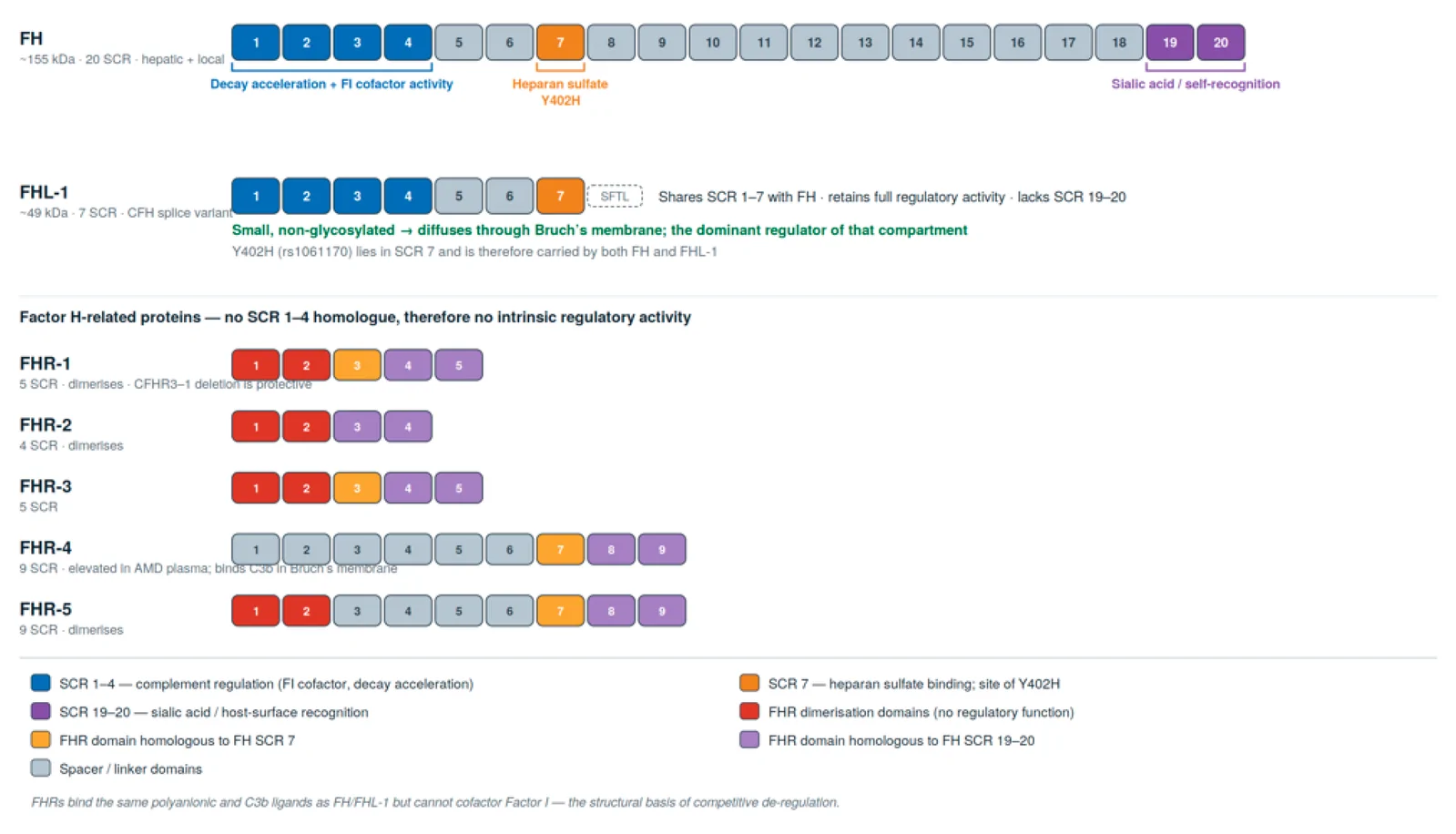
Domain architecture of the Factor H protein family encoded at the RCA locus on chromosome 1q31.3. FH comprises 20 short consensus repeats (SCR); regulatory function resides entirely in SCR 1–4, host surface anchoring in SCR 7 (heparan sulfate) and SCR 19–20 (sialic acid). FHL-1 is the alternatively spliced product of the same gene and consists of SCR 1–7 followed by a four-residue SFTL tail; it retains full regulatory activity but must anchor through SCR 7 alone. The Y402H polymorphism lies within SCR 7 and is therefore carried by both proteins. The FHR proteins possess domains homologous to the ligand-binding regions of FH but lack any counterpart of SCR 1–4, and consequently bind the same surfaces without providing regulatory activity—the structural basis of competitive de-regulation.

The biological functions of FH are segregated into specific functional zones across these 20 domains (Figure 1):

- **SCR 1–4:** These four N-terminal domains contain all the necessary enzymatic regulatory capabilities [12]. They physically bind to C3b and act as an essential cofactor for the serine protease Factor I (CFI). Factor I requires this cofactor assistance to efficiently cleave the active C3b molecule into its inactive form, iC3b, thereby permanently halting its ability to participate in the convertase [6]. Furthermore, SCR 1–4 exert decay-accelerating activity; they physically compete with and displace Factor B (in the form of Bb) from existing C3bBb complexes, actively dismantling the convertase and shutting down the amplification loop [7].
- **SCR 7:** This domain acts as a critical primary anchoring mechanism. It contains a polyanion-binding site that interacts with specific glycosaminoglycans (GAGs), predominantly heparan sulfate (HS), which are abundant in human extracellular matrices and on cell surfaces [6]. This allows FH to tether itself to the host tissues it needs to protect.
- **SCR 19–20:** These C-terminal domains contain a secondary, highly specific surface recognition site that binds to sialic acid and highly sulfated GAGs [6]. Because sialic acid is a ubiquitous marker of host cell membranes but is generally absent from the surfaces of pathogenic bacteria, SCR 19–20 enable FH to differentiate between “self” (which must be protected) and “non-self” (which must be destroyed) [8].

Factor H-like protein 1 (FHL-1), in stark contrast, is a much smaller, 51 kDa protein consisting of only 449 amino acids [6,13]. It is composed entirely of the first seven N-terminal SCR domains of FH (SCR 1 through 7) [12]. Because the first nine exons of the *CFH* transcript are identical for both proteins, FHL-1 shares exact sequence homology with the N-terminus of FH [13]. However, alternative splicing into exon 10 introduces a premature stop codon, terminating FHL-1 with a unique four-amino-acid C-terminal sequence (Ser-Phe-Thr-Leu, or SFTL) and entirely omitting SCR domains 8 through 20 [6]. Remarkably, while FHL-1 is highly expressed in humans, there is no evidence for an alternative *Cfh* splice variant encoding FHL-1 in mice [12]. Rodents possess only the full-length FH protein, rendering murine models of AMD inherently limited in their ability to accurately reflect human complement regulation at the retinal interface [13].

Because FHL-1 retains SCR 1–4, it possesses all the necessary biochemical machinery for robust complement regulation. Extensive serum assays confirm that FHL-1 exhibits potent decay-accelerating activity and acts efficiently as a cofactor for Factor I, demonstrating comparable regulatory activity to full-length FH in protecting host cells from MAC deposition [12]. However, because FHL-1 entirely lacks SCR 19–20, it is devoid of the primary sialic acid host-recognition site [8]. Consequently, FHL-1 cannot rely on sialic acid for tissue targeting; it must rely exclusively on the interactions mediated by SCR 7 [4]. This renders FHL-1’s surface interaction more restricted than that of full-length FH, but critically anchors it to the specific polyanionic signatures of local microenvironments [4].

## 7. Spatial Partitioning in the Retinal Microenvironment

The stark physical differences between FH and FHL-1—specifically regarding molecular weight, glycosylation, and domain structure—dictate their differential spatial distribution and functional supremacy within the distinct ocular compartments. While FH and FHL-1 are reported to exist in equimolar concentrations in the vitreous humor of the eye, their ratio in systemic human plasma is heavily skewed, with FH present at approximately 300 µg/mL and FHL-1 at ~50 µg/mL (a molar ratio of roughly 2:1 when accounting for molecular weight) [15].

The large size (~150 kDa) and extensive glycosylation of full-length FH severely restrict its ability to passively diffuse from the highly fenestrated choroidal vasculature across the dense, lipid-rich, and heavily cross-linked layers of Bruch’s membrane [15]. In contrast, the smaller, 51 kDa, unglycosylated FHL-1 protein easily diffuses out of the choroidal blood stream, permeating deep into the structure of Bruch’s membrane [15].

Sophisticated immunohistochemical mapping and biochemical analyses of human donor eyes have demonstrated spatial partitioning of these two regulatory proteins. Full-length FH is the predominant form protecting the extracellular matrix of the choroid [15]. Furthermore, FH has been shown to coat the outer periphery of large drusen, effectively shielding these inflammatory deposits from the natural immune clearance pathways of the complement cascade, allowing them to persist and grow [15].

Conversely, FHL-1 is the predominant, and likely indispensable, regulator within Bruch’s membrane itself [15]. FHL-1 completely permeates Bruch’s membrane and is heavily immobilized there, largely through specific electrostatic interactions with heparan sulfate via its SCR 7 domain [15]. Furthermore, FHL-1 has been detected deep inside drusen deposits, indicating its intimate involvement in the earliest stages of sub-RPE pathogenesis [16]. This spatial partitioning identifies FHL-1 as the principal regulator of the RPE–Bruch’s membrane interface [16]. If the function of FHL-1 is compromised, this critical anatomical boundary becomes exposed to unregulated amplification of the alternative pathway, paving the way for geographic atrophy.

## 8. The Pathophysiology of Geographic Atrophy: The Y402H Polymorphism

The mechanistic linkage between the failure of the FHL-1/CFH regulatory axis and the onset of geographic atrophy is best illustrated by the Y402H polymorphism (rs1061170). This common, heavily studied missense mutation involves a single nucleotide substitution in exon 9 of the *CFH* gene, where a thymine (T) is replaced by a cytosine (C) at nucleotide position 1277 [6]. This seemingly minor genomic alteration results in the substitution of a neutral tyrosine (Y) amino acid with a positively charged histidine (H) at amino acid position 402 within the resulting protein sequence [6].

Because this mutation occurs within the genetic sequence coding for SCR 7, the defect is transcribed and translated into *both* the full-length FH protein and the truncated FHL-1 variant [17]. The magnitude of the association between the Y402H variant and AMD is large, ranking among the strongest genetic effect sizes ever identified for a complex, multifactorial human disease [9]. Individuals carrying a single copy of the risk allele (heterozygous TC) face a significantly elevated risk of developing AMD [1]. However, individuals homozygous for the risk allele (CC) face up to a sevenfold increased risk compared to the wild-type (TT) population, and present with a significantly earlier age of disease onset [1]. Epidemiological data suggest that the Y402H variant alone accounts for approximately 43% to 50% of the population-attributable risk for developing AMD and progressing to severe geographic atrophy [5,6].

The underlying pathophysiology driven by the Y402H variant is fundamentally a failure of precise spatial targeting, rather than a failure of inherent enzymatic regulation. In vitro assays confirm that the 402H variant retains entirely normal complement-regulatory activity in the fluid phase, as the regulatory domains (SCR 1–4) are completely unaffected by the mutation [4]. However, the histidine substitution fundamentally alters the electrostatic landscape of SCR 7, dramatically reducing the protein’s binding affinity for the specific polyanionic ligands crucial for host surface recognition [11].

Specifically, the Y402H variant severely impairs the binding of FHL-1 to:

1. **Heparan Sulfate** (**HS**)**:** This is the primary, highly sulfated glycosaminoglycan responsible for anchoring FHL-1 to the acellular matrix of Bruch’s membrane and the basolateral surface of the RPE [15].
2. **Oxidized Lipids:** Such as malondialdehyde (MDA). These highly reactive lipid peroxidation products accumulate heavily in the aging retina as a consequence of the oxidative stress generated by the visual cycle. Wild-type FHL-1 binds these toxic lipids to mitigate their pro-inflammatory effects; the mutant fails to do so [11].
3. **Inflammatory Mediators:** Such as C-reactive protein (CRP), a pentraxin intricately involved in the immune clearance of necrotic cellular debris [11].

By compromising the glycosaminoglycan-mediated interaction, the Y402H variant prevents FHL-1 from adequately docking onto Bruch’s membrane and the RPE/retinal interface [4]. Consequently, FHL-1 cannot efficiently localize to the exact sites where C3b deposition is occurring. It is stripped of its ability to act as a cofactor for Factor I directly on the vulnerable tissue surface [17].

## 9. The Biochemical Cascade of Retinal Degeneration

The loss of localized FHL-1 anchoring initiates a self-sustaining biochemical cascade that progressively destroys the macula. First, the ubiquitous “tick-over” of C3 continues unabated within the choroidal and retinal interstitial fluids [7]. As C3b is generated and spontaneously binds to the unprotected surfaces of Bruch’s membrane, it encounters no local regulatory resistance [17]. Factor B binds to the deposited C3b, is cleaved by Factor D, and forms the active C3 convertase (C3bBb) without the decay-accelerating interference normally provided by immobilized FHL-1 [6].

The AP amplification loop proceeds unchecked, causing continuous local deposition of C3b on Bruch’s membrane and the basolateral RPE [16]. This unchecked convertase activity yields exceptionally high local concentrations of the soluble anaphylatoxins C3a and C5a [1]. These potent chemoattractants signal distress to the systemic immune system, recruiting blood-derived monocytes, macrophages, and resident retinal microglia into the subretinal space [5]. What began as necessary parainflammation—an attempt to clear accumulated debris and restore homeostasis—escalates into chronic, destructive, macrophage-mediated inflammation [5].

Simultaneously, the unchecked C3b deposition inevitably leads to the formation of the C5 convertase, which rapidly cleaves C5 into C5b [5]. This triggers the terminal complement pathway, leading to the assembly of the Membrane Attack Complex (MAC) directly on the lipid bilayers of the RPE cells [5]. High-resolution proteomic and immunohistochemical analyses of donor eyes confirm the dense accumulation of C3, C5, and MAC components within drusen and directly on the RPE of AMD patients [5].

Over years of enduring chronic low-level inflammation, oxidative damage, and the constant osmotic stress induced by MAC insertion, the RPE cells are driven toward apoptosis and necrosis [15]. Because the RPE is essential for the metabolic support and survival of the neuroretina, the death of the RPE inevitably and irreversibly triggers the secondary death of the overlying photoreceptors [1]. This expanding front of combined RPE and photoreceptor death is the direct cellular mechanism underlying the progressive, blinding lesions observed clinically as geographic atrophy [1].

## 10. The Competitive Antagonists: Factor H-Related Proteins (FHRs)

The regulation of the alternative pathway is not solely determined by the presence or absence of functional FH and FHL-1. It is further complicated by a secondary layer of modulation provided by the Factor H-Related (FHR) proteins. The RCA gene cluster on chromosome 1q32 encodes five distinct FHR proteins (designated FHR-1 through FHR-5) [18]. These proteins are the result of relatively recent evolutionary gene duplication events of the ancestral *CFH* gene [18]. Notably, these duplication events occurred late in mammalian evolution, specifically after the phylogenetic divergence of the rodent and primate lineages [18]. Consequently, mice lack these specific *CFHR* genes, further illustrating why murine models fail to capture the full complexity of human AMD pathogenesis and why human-specific targeted therapies are required [18].

Structurally, FHR proteins are composed entirely of SCR domains that are highly homologous to the ligand-binding C-terminal domains of full-length FH (such as SCR 7 and SCR 19–20) [7]. Therefore, FHRs possess a robust capacity to bind to host tissues, heparan sulfate, sialic acid, and deposited C3b [19]. However, critically, the evolutionary duplication events did not include the N-terminal domains. The FHR proteins completely lack domains homologous to SCR 1–4 of FH/FHL-1 [11]. As a direct result, FHRs possess no intrinsic complement regulatory capability; they cannot act as a cofactor for Factor I, nor can they accelerate the decay of the C3 convertase.

In the pathophysiology of geographic atrophy, FHR proteins act as competitive antagonists to the protective FH and FHL-1 proteins. Clinical studies have consistently demonstrated that elevated systemic circulating levels of FHR-1, FHR-4, and FHR-5 are strongly, independently correlated with an increased risk of developing advanced AMD [20].

The mechanism is driven by competitive displacement. FHR-1 and FHR-5 effectively compete with FH and FHL-1 for identical binding sites on extracellular matrices, exposed DNA, and the surfaces of apoptotic or necrotic cells [20]. FHR proteins frequently form homodimers or heterodimers, significantly enhancing their avidity for target surfaces [20]. When an FHR-1 dimer binds to C3b or heparan sulfate on Bruch’s membrane, its high avidity allows it to physically displace any associated FH or FHL-1 molecules [19]. Because the displacing FHR lacks the regulatory SCR 1–4 domains, the displaced FHL-1 can no longer assist Factor I in cleaving C3b [20]. The surface-bound C3b remains active, freely binding Factor B to form a durable C3bBb convertase that continues to amplify the AP cascade despite the presence of FHL-1 in the interstitial fluid [20].

Furthermore, FHR-1 actively drives cellular inflammation beyond simple complement amplification. Histological studies show that FHR-1 accumulates heavily below the RPE in AMD donor tissues. In this sub-RPE space, FHR-1 binds to specific receptors (such as Emr1) on invading mononuclear phagocytes and resident RPE cells [21]. This interaction triggers intracellular calcium signaling and alters gene expression, promoting a chronic pro-inflammatory state that accelerates tissue degeneration [21].

Conversely, genetic studies indicate that the chromosomal deletion of the *CFHR1* gene (and often the adjacent *CFHR3* gene) is associated with a reduced risk of AMD [21]. By completely removing this competitive antagonist from the system, the remaining FH and FHL-1 proteins face no competition for tissue binding sites, allowing them to exert maximal regulatory control. The presence of FHR proteins therefore acts as a critical biological rheostat, continuously adjusting the delicate balance between complement activation and regulation on host surfaces [21]. In the context of a patient carrying the functionally compromised Y402H FHL-1 variant, the competitive antagonism exerted by normal or elevated levels of FHR proteins is predicted to shift this balance further toward unregulated complement amplification.

### Grading the Evidence Implicating FHR Proteins

The evidence implicating the FHR proteins in AMD is not of uniform strength, and conflating its tiers has produced avoidable overstatement in this field. Four levels should be distinguished, and the therapeutic arguments that follow rest on different levels for different targets.

**Genetic association.** This is the strongest tier. The common deletion of *CFHR3* and *CFHR1* is reproducibly associated with reduced AMD risk across independent cohorts, and the strongest AMD-associated variant at the *CFH* locus, rs10922109, is associated with lower FHR-4 concentrations independently of that deletion [19,20]. These are replicated observations in large samples. They are nonetheless associations at a locus in which several genes lie in linkage disequilibrium, and they do not by themselves identify which gene product is causal.

**Circulating protein association.** Plasma FHR-4 is elevated in AMD at high statistical confidence whereas plasma FH is not, and raised FHR-1 and FHR-5 have also been reported in advanced disease [19,20]. This tier connects genotype to a measurable protein phenotype. The studies are, however, cross-sectional and cannot establish the direction of causality, and systemic concentration is an imperfect proxy for concentration within Bruch’s membrane, which is the compartment that matters here.

**Tissue-level evidence.** FHR-4 has been localized to the choriocapillaris, Bruch’s membrane and drusen in human donor eyes, and FHR-1 accumulates in the sub-RPE space in AMD tissue, where it engages receptors on mononuclear phagocytes and alters their signaling [14,19,21]. In vitro, FHR-4 competes with FH and FHL-1 for C3b and prevents Factor I-mediated C3b cleavage [19]. This tier supplies the mechanism, and it is the tier on which the therapeutic rationale developed below principally rests. Donor-eye studies are nevertheless observational and cannot separate cause from consequence in tissue where atrophy is already established.

**Causal and interventional evidence.** This tier is currently empty. No FHR protein has been targeted in a clinical trial in geographic atrophy; no interventional data exist for FHR reduction in any ocular indication; and no Mendelian randomization analysis has established a causal effect of FHR-4 concentration on lesion growth. The proposal that FHR silencing would be therapeutically useful is therefore a mechanistic hypothesis supported by genetic and tissue observation, and should be read as such—particularly in a field where both lampalizumab and *CFI* gene augmentation failed after preclinical rationale of comparable apparent strength [22,23].

## 11. Complement Inhibition in Current Clinical Practice

Two intravitreal complement inhibitors are approved in the United States for geographic atrophy secondary to AMD. Both act on the cascade itself rather than on its regulators, and both are summarized here only so far as is needed to frame the RNA interference question; detailed appraisals of their trial data, health-economic profile, and real-world use are available elsewhere [24].

Pegcetacoplan is a pegylated pentadecapeptide that binds C3 and C3b, acting at the point of convergence of all three activation pathways. In the phase 3 OAKS and DERBY trials, it reduced growth of the GA lesion area relative to sham, and the effect increased with continued treatment in the GALE open-label extension, reaching a reduction of up to 32% across all eyes at 36 months with larger effects in non-subfoveal lesions [25,26]. Best-corrected visual acuity did not separate from sham over the randomized period; a prespecified microperimetry analysis at 36 months showed 18% fewer new scotomatous points with monthly treatment [26]. It was approved by the FDA in February 2023. The European Medicines Agency did not grant marketing authorization.

Avacincaptad pegol is a pegylated RNA aptamer that binds C5 and prevents its cleavage into C5a and C5b. In GATHER1 and GATHER2 it reduced GA lesion growth relative to sham over 12 months, with the treatment effect increasing through the second year of GATHER2, and it was approved by the FDA in August 2023 [27,28]. GATHER2 excluded lesions involving the foveal center point whereas OAKS and DERBY did not, so the two programs are not directly comparable.

Three features of this approved class bear directly on what follows. First, both agents slow lesion growth without a demonstrated benefit in visual acuity over the randomized period, and approval rests on an anatomical endpoint. Second, both require monthly or every-other-month intravitreal injection indefinitely, which is a considerable burden in an elderly population. Third, both carry class adverse effects: new-onset exudative AMD occurred in approximately 5–7% of monthly-treated patients in the pivotal trials, and post-marketing surveillance of pegcetacoplan has identified occlusive and non-occlusive retinal vasculitis at an estimated rate of about one per 4000 first injections, alongside rare ischemic optic neuropathy [26,29]. Treatment durability, route of administration, and the consequences of broad cascade suppression are precisely the pressures that motivate interest in nucleic acid approaches with long dosing intervals and narrowly defined molecular targets.

The class also has a substantial record of failure, which constrains how confidently any mechanistic argument should be advanced. Lampalizumab, an antibody fragment directed at Factor D, produced an encouraging phase 2 signal but showed no effect on lesion growth in the phase 3 CHROMA and SPECTRI trials—the largest GA studies conducted to that point—including within the biomarker-selected subgroup [22]. Subretinal AAV-mediated *CFI* augmentation (GT005/PPY988) was discontinued in 2023 after an independent data monitoring committee concluded that the futility criteria had been met [23]. That genetic validation of a target has repeatedly failed to translate into clinical benefit is a caution against inferring efficacy from mechanism, and it applies with equal force to the strategies discussed below.

## 12. RNA Interference (siRNA) Therapeutics: Mechanisms, Evolution, and Delivery

The recognition that geographic atrophy is driven in part by the genetic overproduction of pathogenic complement amplifiers (like C3 and C5), the presence of competitive antagonists (like FHR-1), and the functional insufficiency of localized regulators (like FHL-1) has spurred intensive pharmaceutical research into targeted gene-silencing technologies. The most prominent and clinically advanced of these modalities is RNA interference (RNAi) [30].

RNA interference is a highly conserved, endogenous post-transcriptional gene silencing pathway utilized by eukaryotic cells to regulate gene expression and defend against double-stranded RNA viruses [30]. Small interfering RNAs (siRNAs) are synthetic, double-stranded RNA molecules, typically 19 to 23 nucleotides in length, meticulously engineered to harness this natural intracellular machinery for therapeutic benefit [31].

Upon successful delivery into the cytoplasm of a target cell, the synthetic siRNA duplex engages the RNAi pathway. While endogenous long double-stranded RNA must first be cleaved by the RNase III enzyme Dicer, modern synthetic siRNAs are designed to bypass this step entirely [31]. The siRNA is directly incorporated into a multi-protein complex known as the RNA-induced silencing complex (RISC) [31]. Within RISC, the duplex is unwound; the passenger (sense) strand is cleaved and discarded, while the guide (antisense) strand remains securely bound [32]. The guide strand directs the RISC complex to scan the cellular cytoplasm for messenger RNA (mRNA) transcripts exhibiting strict, Watson–Crick sequence complementarity to the guide sequence [32]. Once the target mRNA is identified and bound, the argonaute 2 (Ago2) endonuclease component of RISC catalyzes the cleavage of the target mRNA, initiating its rapid degradation by cellular exonucleases and entirely preventing its translation into a pathogenic protein [32].

The pharmacological potency of siRNA is high. Because a single RISC complex can remain active for weeks and sequentially cleave hundreds of target mRNA transcripts, siRNA exhibits a highly catalytic, durable mechanism of action that far exceeds the stoichiometric limitations of traditional small molecule inhibitors or monoclonal antibodies [30].

## 13. Overcoming Biological Barriers: Chemical Modification and GalNAc Conjugation

The initial clinical translation of siRNA therapeutics was limited by several physiological barriers. “Naked,” unmodified siRNAs are highly vulnerable to rapid degradation by ubiquitous serum endonucleases, are easily eliminated by renal filtration due to their small size and polyanionic charge, and are incapable of passively crossing the lipophilic cell membrane [33]. Furthermore, exogenous double-stranded RNA can be recognized by the innate immune system (specifically Toll-like receptors, such as TLR3) as a viral signature, triggering a potentially severe interferon response [33].

These barriers have been substantially overcome through two decades of sophisticated biochemical engineering. Modern siRNA therapeutics utilize extensive chemical modifications—such as 2′-O-methyl and 2′-fluoro substitutions on the ribose sugar backbone, and phosphorothioate linkages between nucleotides—to vastly enhance nuclease resistance, increase binding affinity to the target mRNA, and entirely abrogate immunogenic TLR activation [34].

Furthermore, the issue of targeted cellular delivery has been transformed by the development of specific ligand conjugates. The most successful of these is N-acetylgalactosamine (GalNAc) [30]. GalNAc is a sugar molecule that binds with exceptional affinity to the asialoglycoprotein receptor (ASGPR), a receptor heavily and almost exclusively expressed on the surfaces of hepatocytes (liver cells) [30]. By covalently conjugating a tri-antennary GalNAc ligand to the siRNA duplex, the therapeutic can be administered systemically via a simple subcutaneous injection [30]. The GalNAc moiety guides the siRNA directly to the liver, where it is rapidly internalized by receptor-mediated endocytosis, allowing the siRNA to escape into the cytoplasm and engage RISC efficiently [30].

Given that the liver is the primary biosynthetic organ for the vast majority of circulating plasma complement proteins—including C3, C5, Factor B, and systemic Complement Factor H—the GalNAc-siRNA delivery platform represents an exceptionally potent, durable, and targeted tool for systemic complement modulation [30]. While hepatic targeting dominates the current landscape, extra-hepatic delivery of siRNA is rapidly advancing. These advances signal a near-future capability where siRNA could be systemically administered and specifically targeted to receptors on the RPE or choroidal endothelium, circumventing the need for hepatic intermediaries.

## 14. Directly Addressing the Core Question: Can siRNA Target the FHL-1 Pathway?

Given the protective role of FHL-1 on Bruch’s membrane, a critical therapeutic question emerges: Can siRNA therapy be utilized to directly target the FHL-1 pathway to treat geographic atrophy?

The answer to this question is highly nuanced, requiring a strict biochemical differentiation between targeting a *gene* and targeting a *pathway*. The direct application of a conventional siRNA molecule to simply knock down the expression of the *CFH* gene (and thus eliminate FHL-1) would be expected to worsen disease in a patient with geographic atrophy.

## 15. Why Direct FHL-1 Silencing Is Contraindicated

FHL-1 is fundamentally a protective regulatory protein [7]. Geographic atrophy is driven by the insufficiency or dysfunction of FHL-1 (as seen in the Y402H polymorphism or in patients with early-onset macular drusen who exhibit a ~50% decrease in FHL-1 expression), not its overactivity [17]. The entire genetic architecture of AMD indicates that the loss of FH/FHL-1 regulatory function is the direct cause of uncontrolled MAC deposition and RPE cell death [12].

This genetic epidemiological inference is supported by in vitro knockdown models. When human immortalized choroidal endothelial cells (CECs) or human RPE cell lines are transfected with *CFH*-specific siRNA to artificially knock down the endogenous expression of FH and FHL-1, the consequences are pathogenic [15]. Cells treated with *CFH* siRNA exhibit a statistically significant increase in Membrane Attack Complex (MAC) deposition on their surfaces upon exposure to human serum [15]. Furthermore, the siRNA-mediated knockdown of *CFH* triggers the robust activation of the NF-κB inflammatory signaling pathway within RPE cells, demonstrating that the loss of FHL-1 directly incites intracellular stress and inflammatory cytokine production [11].

Therefore, designing an siRNA drug intended to globally suppress FHL-1 expression is contraindicated. It would be expected to remove the remaining regulatory shielding from Bruch’s membrane, vastly accelerating the progression of geographic atrophy [15]. FHL-1 is the principal regulator at this interface, and its removal would be expected to accelerate disease.

## 16. Indirect Therapeutic Strategies Utilizing siRNA in the FHL-1 Axis (Figure 2)

While pan-silencing of FHL-1 is demonstrably harmful, the sophisticated, sequence-specific capabilities of RNA interference offer several highly targeted, indirect approaches to correct the complement regulatory imbalance and support the FHL-1 pathway.

**Figure 2:**
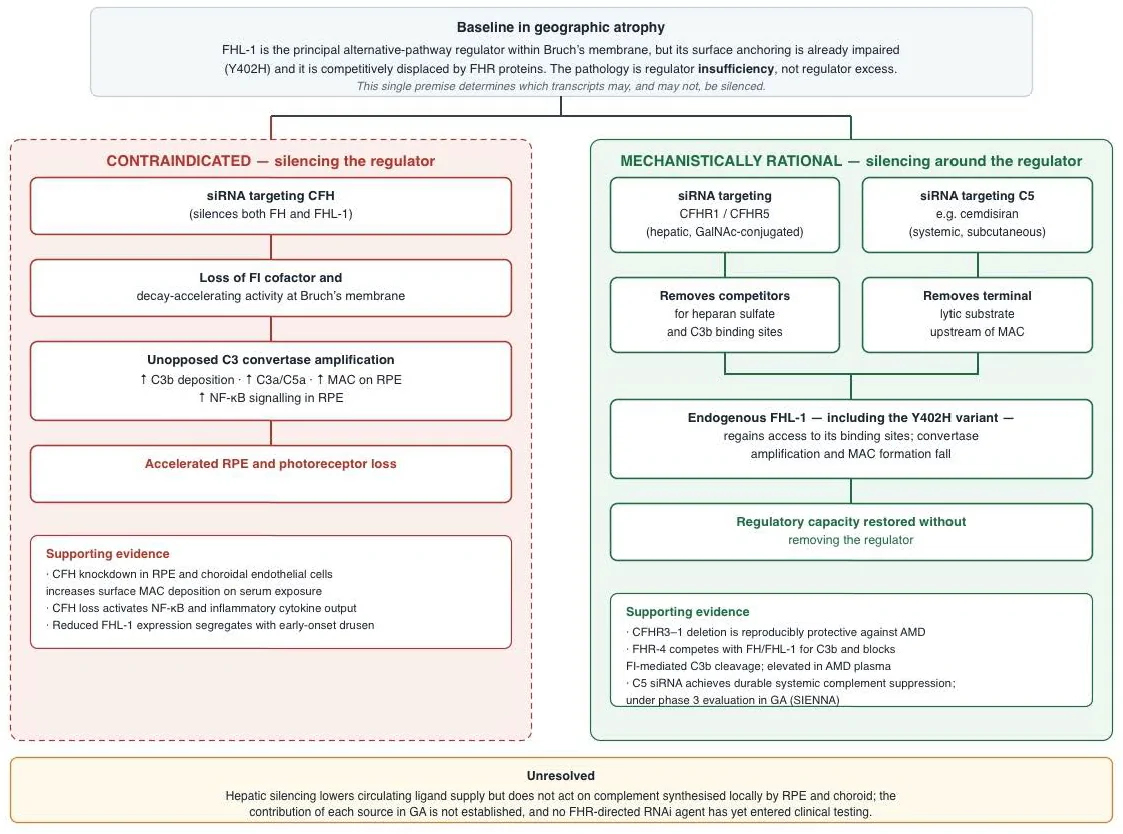
Therapeutic logic of RNA interference within the FHL-1/CFH axis. Because the pathology is one of regulator insufficiency rather than regulator excess, silencing *CFH* removes the very activity that is already deficient and would be expected to accelerate atrophy (**left**). Silencing around the regulator—removing the competing FHR proteins, or removing the terminal substrate C5—shifts the binding equilibrium at Bruch’s membrane in favor of the endogenous regulator that remains, including the poorly anchoring Y402H variant (**right**). The distinction is between targeting a gene and targeting a pathway. The caveats shown are material: hepatic silencing does not act on complement synthesized locally by RPE and choroid, and no FHR-directed RNAi agent has yet entered clinical testing.

### 16.1. Allele-Specific Silencing of the Y402H Variant

A highly advanced, theoretical application of RNAi is allele-specific silencing. Because siRNA relies on strict Watson–Crick base pairing to identify its target mRNA, it is theoretically possible to design an siRNA guide strand that perfectly matches the mutant mRNA sequence containing the single nucleotide substitution (rs1061170, T>C) responsible for the Y402H variant, but contains a central mismatch for the wild-type sequence [5].

In a heterozygous patient (TC genotype), an allele-specific siRNA could selectively direct the RISC complex to degrade only the mutant mRNA transcripts encoding the defective 402H FHL-1 protein while leaving the wild-type 402Y transcripts untouched and fully functional. By eliminating the defective protein from the local microenvironment, the competitive inhibition and pathological aggregation it exerts on the wild-type protein is removed. This approach would theoretically restore a normalized, highly functional regulatory environment on Bruch’s membrane. However, to prevent total complement dysregulation, this approach would likely be restricted to heterozygotes whose remaining wild-type allele can sufficiently upregulate expression to compensate for the loss of half their total FHL-1 transcript volume.

### 16.2. Silencing the Competitive Antagonists: Targeted Knockdown of FHR-1 and FHR-5

The most mechanistically coherent application of siRNA related to the FHL-1 pathway is the targeted knockdown of the Factor H-Related proteins, specifically FHR-1 and FHR-5.

As established previously, FHR-1 and FHR-5 act as potent competitive antagonists; they bind with high avidity to host tissue surfaces and physically block FHL-1 from docking to heparan sulfate, without providing any complement regulatory activity to stop the C3 convertase [21]. Genetic data indicate that the deletion of CFHR1 and CFHR3 is associated with reduced AMD risk, although this confers partial rather than complete protection [21].

By deploying a GalNAc-conjugated siRNA designed to selectively silence the hepatic expression of the *CFHR1* or *CFHR5* genes, the circulating levels of these antagonistic proteins would plummet [21]. In the absence of FHR-1 and FHR-5 in the choroidal circulation, even the weakened Y402H variant of FHL-1 would face zero competition for the critical heparan sulfate binding sites on Bruch’s membrane. This “derepression” strategy leverages siRNA not to attack the primary regulator, but to eliminate its competitor. By doing so, it indirectly restores the functional dominance of FHL-1 at the RPE/Bruch’s membrane interface [21]. Extensive patent literature indicates that major pharmaceutical entities are pursuing this strategy, filing patents detailing the use of siRNA, shRNA, or antisense oligonucleotides specifically designed to reduce the levels of FHR-1, FHR-2, and FHR-5 to restore AP balance in geographic atrophy [21].

### 16.3. Dual-Targeting siRNAs (Bispecific siRNA)

The biological complexity of complement regulation is highly amenable to emerging “bispecific siRNA” technologies [35], which are engineered to silence two independent genetic targets simultaneously through a single molecular construct. The traditional “one gene, one disease” approach often fails in highly networked systems like complement immunity [36].

A bispecific siRNA could be designed to simultaneously silence a downstream terminal effector (such as C5) and a competitive antagonist (such as FHR-1). This highly synergistic approach would suppress the terminal lytic machinery (MAC formation) while simultaneously favoring FHL-1-mediated regulation at the level of the C3 convertase by removing its antagonist. Such combinatorial approaches represent the cutting edge of nucleic acid therapeutics for complex genetic diseases [21].

## 17. Alternative Strategies: Augmenting Rather than Silencing the Regulatory Arm

RNA interference can reduce the abundance of a transcript; it cannot increase one. Because the defect in the FHL-1/CFH axis is one of insufficient or mislocalized regulatory activity, approaches that raise functional regulator levels are conceptually the more direct correction, and they merit consideration alongside the silencing strategies described above. Reviewer attention to this point is well placed: an account of this axis that discussed only gene silencing would misrepresent the therapeutic landscape.

### 17.1. Gene Augmentation

The most clinically advanced of these approaches is AAV-mediated gene augmentation. Subretinal delivery of *CFI* (GT005/PPY988) reached phase 2 before discontinuation, and ocular biomarker data from the phase 1/2 FOCUS study demonstrated measurable intraocular increases in Factor I with corresponding shifts in complement breakdown products, confirming that local augmentation of a soluble regulator is achievable even though clinical benefit was not demonstrated [23]. More directly relevant to the present argument, Grigsby and colleagues compared AAV constructs expressing human FHL-1 and a truncated *CFH* retaining the functional N- and C-terminal domains in *Cfh*-null mice and found that the two constructs differentially restored complement regulation in the eye versus the plasma compartment [10]. That result is instructive: FHL-1 and truncated FH are not interchangeable, and the compartment in which regulation is restored depends on which construct and which route of delivery is used.

Full-length *CFH* approaches the packaging limit of AAV, which is one reason that truncated constructs have been pursued—including FHL-1 itself and engineered ‘mini-FH’ proteins that fuse the SCR 1–4 regulatory module directly to the SCR 19–20 surface-recognition module. FHL-1 is an attractive payload for the macula specifically: it is small, unglycosylated, and diffuses through Bruch’s membrane, which is where the regulatory deficit lies [16,37]. A construct expressing wild-type 402Y FHL-1 in the RPE would in principle supply correctly anchoring regulator directly into the compartment that a Y402H homozygote cannot adequately protect, and would do so without the need to reduce any transcript.

### 17.2. Protein Supplementation and Transcriptional Approaches

Several other routes to the same end have been proposed. Recombinant protein supplementation, whether of FHL-1 or of mini-FH, avoids the irreversibility of gene transfer but reintroduces the burden of repeated intravitreal dosing that both RNAi and gene therapy are intended to escape. Transcriptional upregulation of the endogenous *CFH* locus—through CRISPR activation, or through modulation of the inflammatory signaling that governs *CFH* expression in RPE—remains preclinical, and carries a specific logical difficulty: in a Y402H carrier, it would simply produce more of a protein that anchors poorly. For that reason, allele-selective correction, whether via the base editing of rs1061170 or by the allele-specific silencing described earlier, is mechanistically more coherent than any strategy that raises the total *CFH* output indiscriminately.

Augmentation and silencing are complementary rather than competing. Restoring regulator activity locally and removing competing FHR proteins systemically act on opposite sides of the same binding equilibrium at Bruch’s membrane, and there is no pharmacological reason why the two could not be combined. Which is appropriate for a given patient is likely to depend on genotype, as considered in the closing section. It should be stated plainly, however, that the single clinical test of ocular complement augmentation conducted to date did not succeed, and the reasons for that failure—whether target choice, expression level, timing relative to established atrophy, or the anatomical endpoint itself—remain unresolved [23].

### 17.3. Limitations

Several limitations of this review should be stated. It is narrative rather than systematic, and article selection reflects the author’s judgement of relevance rather than a prespecified appraisal protocol. Its scope is deliberately narrow, and it does not attempt a balanced survey of non-complement mechanisms in geographic atrophy, of neuroprotective or cell-replacement approaches, or of the wider therapeutic pipeline. Much of the mechanistic evidence for the spatial argument derives from human donor tissue, which is cross-sectional and taken from eyes in which atrophy is already established. The rodent models most often used lack an FHL-1 orthologue and lack the *CFHR* gene family altogether, so the animal literature cannot arbitrate the questions raised here [18]. Finally, the therapeutic proposals discussed—allele-specific silencing, FHR knockdown and dual-targeting constructs—have not been tested clinically in this indication, and the record of complement-directed agents in geographic atrophy counsels against treating mechanistic plausibility as a predictor of benefit.

## 18. Synthesis and Strategic Conclusions

Geographic atrophy represents the terminal, irreversibly blinding phase of a decades-long immune-mediated conflict occurring at the critical anatomical interface of the retinal pigment epithelium and Bruch’s membrane. The biochemical, genetic, and spatial data identify the FHL-1 protein—a 51 kDa, truncated, highly permeable, non-glycosylated alternative splice variant of Complement Factor H—as the indispensable, primary biological regulator of Bruch’s membrane against unchecked amplification of the alternative complement pathway [12]. The pervasive Y402H polymorphism severely and specifically compromises the ability of FHL-1 to anchor to heparan sulfate within the extracellular matrix [15]. This critical failure in spatial localization strips the host tissues of their regulatory shielding, precipitating a self-perpetuating cascade of C3b opsonization, parainflammation, macrophage recruitment, and ultimate Membrane Attack Complex deposition on the RPE [5].

The application of small interfering RNA (siRNA) to this highly complex pathophysiological matrix requires acute mechanistic precision. Direct suppression of the FHL-1 transcript via conventional siRNA is fundamentally counterproductive and pro-inflammatory, as demonstrated unequivocally by the exacerbation of MAC deposition and NF-κB activation observed in in vitro knockdown studies [11]. FHL-1 constrains alternative pathway activity at this interface, and reducing it would be expected to accelerate degeneration.

Instead, siRNA therapeutics must be deployed laterally to strategically alter the overall stoichiometry of the complement cascade. Systemic suppression of the terminal effector C5 using advanced agents like cemdisiran provides a potent, durable blockade of the terminal lytic pathway, and is currently demonstrating the viability of systemic RNAi in pivotal Phase 3 trials for geographic atrophy [24]. However, more specifically aligned with the biology of the FHL-1 axis, siRNA targeted against the Factor H-Related proteins (FHR-1 and FHR-5) represents a mechanistically rational therapeutic avenue [21]. By eliminating these competitive antagonists, targeted gene silencing allows the existing, endogenous FHL-1 (even the weakened Y402H variant) to bind to Bruch’s membrane without interference, thereby maximizing its residual regulatory capacity and indirectly restoring complement homeostasis [21].

Ultimately, resolving geographic atrophy will demand interventions capable of crossing the strict structural boundaries of the retina to modulate a highly localized, self-sustaining inflammatory process. The future of GA therapeutics will likely favor a highly personalized matrix of interventions based on high-resolution patient genotyping. A patient homozygous for the Y402H mutation and suffering from severe FHL-1 insufficiency may benefit maximally from an AAV-mediated FHL-1 gene augmentation delivered directly to the subretinal space [6]. Conversely, a patient exhibiting high systemic titers of FHR-1 and elevated C5 might be ideally suited for a systemic, subcutaneous siRNA (like cemdisiran or an FHR-1 targeted siRNA) to shut down the supply of competing antagonists and anaphylatoxins arriving via the choroidal circulation. Whether through the local synthesis of functional regulators, the systemic RNAi-mediated suppression of complement amplifiers, or sophisticated bispecific dual-modality approaches, manipulating the FHL-1/CFH axis remains among the best genetically supported strategies for halting the progression of geographic atrophy.

## Figures and Tables

**Table 1:** Comparative summary of the complement proteins most relevant to geographic atrophy. Evidence is graded by tier—genetic association, circulating protein association, tissue-level observation, and interventional data—so that the strength of support for each proposed target can be read directly. SCR, short consensus repeat; FI, Factor I; MAC, membrane attack complex; RPE, retinal pigment epithelium.

Therapeutic Targeting Status	Evidence Linking to AMD/GA, by Tier	Principal Site of Action in the Macula	Intrinsic Regulatory Activity	Structure	Protein (Gene)
Not a silencing target—loss of function is the pathology. AAV and mini-FH augmentation preclinical.	**Genetic:** Y402H (rs1061170) and rs10922109 among the strongest AMD signals. **Circulating:** plasma FH not elevated in AMD. **Tissue:** localized to choroid and drusen periphery. **Interventional:** none directed at FH.	Choroidal stroma; coats the periphery of large drusen. Diffuses poorly into Bruch’s membrane	Full: FI cofactor and decay acceleration (SCR 1–4); surface recognition (SCR 7 and 19–20)	155 kDa; 20 SCR; glycosylated; mainly hepatic	FH (*CFH*)
Silencing contraindicated. AAV-mediated FHL-1 augmentation preclinical.	**Genetic:** carries Y402H in common with FH. **Circulating:** reduced expression reported in early-onset drusen. **Tissue:** dominant regulator of Bruch’s membrane in donor eyes. **Interventional:** none.	Predominant alternative-pathway regulator within Bruch’s membrane; also detected within drusen	Full regulatory activity (SCR 1–4); anchoring depends on SCR 7 alone	49 kDa; SCR 1–7 plus a four-residue SFTL tail; unglycosylated	FHL-1 (*CFH*, splice variant)
Mechanistically rational silencing target; no clinical-stage agent.	**Genetic:***CFHR3–1* deletion associated with reduced AMD risk (replicated). **Circulating:** raised in advanced AMD. **Tissue:** accumulates below the RPE in AMD donor eyes. **Interventional:** none.	Sub-RPE space; binds C3b and heparan sulfate; engages receptors on mononuclear phagocytes	None—no SCR 1–4 homologue	~38 kDa; 5 SCR; forms homo- and heterodimers	FHR-1 (*CFHR1*)
Mechanistically rational silencing target; no clinical-stage agent.	**Genetic:** rs10922109 strongly associated with FHR-4 level, independent of *CFHR3–1* deletion. **Circulating:** elevated in AMD. **Tissue:** competes with FH/FHL-1 for C3b and blocks FI-mediated cleavage. **Interventional:** none.	Choriocapillaris, Bruch’s membrane and drusen	None	~86 kDa; 9 SCR	FHR-4 (*CFHR4*)
Proposed silencing target; evidence weaker than for FHR-1 and FHR-4.	**Genetic:** locus association at 1q31.3. **Circulating:** raised in advanced AMD. **Tissue:** competitive displacement of FH/FHL-1 shown in vitro. **Interventional:** none.	Extracellular matrix and C3b-bearing surfaces	None	~65 kDa; 9 SCR; dimerizes	FHR-5 (*CFHR5*)
Clinically validated at the anatomical endpoint. Intravitreal peptide approved (FDA 2023); no ocular RNAi program.	**Genetic:***C3* R102G associated with AMD. **Circulating:** activation products raised in AMD. **Tissue:** dense deposition in drusen and on RPE. **Interventional:** pegcetacoplan slowed lesion growth in two phase 3 trials.	Produced systemically and locally by RPE; deposits on Bruch’s membrane and within drusen	Substrate, not a regulator; source of C3a and C3b	185 kDa; central effector of all three pathways	C3 (*C3*)
Clinically validated at the anatomical endpoint. Aptamer approved (FDA 2023); cemdisiran (siRNA) in phase 3 for GA.	**Genetic:** locus signal weaker than at *CFH*. **Circulating:** activation products raised. **Tissue:** MAC demonstrated on RPE in AMD donor eyes. **Interventional:** avacincaptad pegol slowed lesion growth in two phase 3 trials.	MAC assembly on the RPE plasma membrane	Substrate; source of C5a and of C5b for MAC assembly	190 kDa; initiates the terminal pathway	C5 (*C5*)
Target validity not confirmed clinically. The principal cautionary precedent in this field.	**Genetic:** weak locus association. **Circulating:** raised in AMD. **Tissue:** present in drusen. **Interventional:** lampalizumab showed no effect in CHROMA and SPECTRI, including in the biomarker-selected subgroup.	One of the few complement proteins, with FHL-1 and C5a, shown to diffuse freely through Bruch’s membrane	Amplification enzyme; cleaves CFB within the C3bB complex	24 kDa serine protease; constitutively active; rate-limiting for the alternative pathway	Factor D (*CFD*)

## Data Availability

No new data were created or analyzed in this study. Data sharing is not applicable to this article.
